# Endoscopic intraperitoneal subserosal dissection for tumor in the spleen–stomach space

**DOI:** 10.1055/a-2646-1470

**Published:** 2025-07-25

**Authors:** Shao-Bin Luo, Zu-Qiang Liu, Li Wang, Quan-Lin Li, Ping-Hong Zhou

**Affiliations:** 1 92323 Endoscopy Center and Endoscopy Research Institute, Zhongshan Hospital Fudan University, Shanghai, China; 2 Shanghai Collaborative Innovation Center of Endoscopy, Shanghai, China; 3 729313 Endoscopy Center, Shanghai Geriatric Medical Center, Shanghai, China

**Fig. 1:**
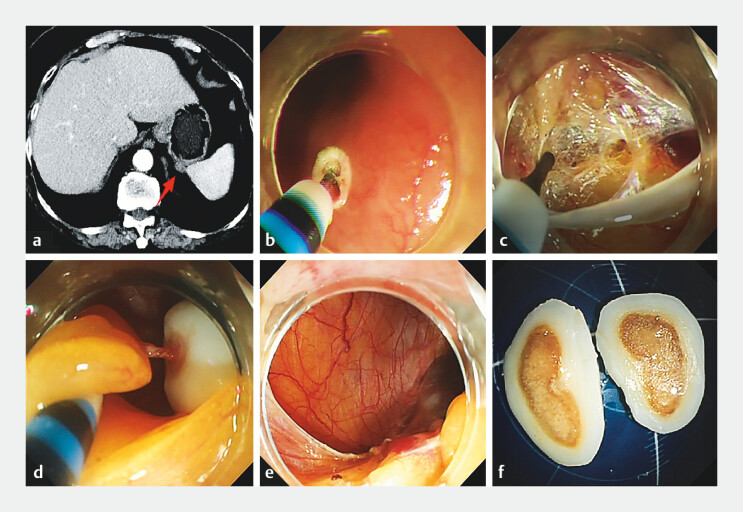
**a** Abdominal CT in a 73-year-old woman admitted with a submucosal tumor in the gastric fundus showed the tumor located in the spleen–stomach space and connected to the gastric fundus mucosa. **b, c** After establishing the submucosal tunnel and dissection of the full thickness of the gastric wall, no obvious mass was found in the gastric wall. **d** Between the spleen and the gastric wall, a mass was found on the greater omentum, connected by a feeding vessel. **e, f** After cauterization of the vessels with hot biopsy forceps, the tumor was completely removed.

A 73-year-old woman was admitted with a submucosal tumor in the gastric fundus. Abdominal CT showed that the tumor was located in the spleen–stomach space and was connected to the gastric fundus mucosa ( Fig. 1 **a** ). Endoscopy showed a mucosal bulge in the gastric fundus. Endoscopic intraperitoneal subserosal dissection (EISD) was performed ( Video 1 ). After establishing the submucosal tunnel and dissection of the full thickness of the gastric wall, no obvious mass was found in the gastric wall, and the gastroscope was then introduced into the abdominal cavity ( Fig. 1 **b, c** ). Between the spleen and the gastric wall, a mass was found on the greater omentum, connected by a feeding vessel ( Fig. 1 **d** ). The mass was dragged into the gastric lumen and was completely removed ( Fig. 1 **e, f** ). The wound was sutured with a metal clip combined with nylon suture thread. The patient was discharged without complication on postoperative day 3. One year after operation, follow-up endoscopy showed no recurrence.

Endoscopic intraperitoneal subserosal dissection for tumor in the spleen–stomach space.Video 1

Given the specific anatomy of the stomach, with its large and nonlinear lumen, nonfixed position, and high flexibility, establishing a submucosal tunnel for submucosal tunneling endoscopic resection is technically challenging. The EISD procedure boasts notable advantages. Firstly, the intact mucosa at the lesion site and the short tunnel can reduce infections and other complications resulting from full-thickness perforations. Second, compared with the uneven full-thickness defects caused by endoscopic full-thickness resection (EFTR), the tunnel makes it easier to close the mucosal defect. Importantly, the distance between the perforation and the lesion allows direct, full exposure of the lesion from the abdominal cavity, rather than from a tangential view within the gastric cavity or submucosal tunnel. This is a crucial safety factor for precise dissection and adequate hemostasis. EISD may be a safe and effective technique for the management of tumors in the abdominal cavity close to the stomach wall.

Endoscopy_UCTN_Code_TTT_1AO_2AG_3AF

